# Plant-based dietary patterns defined by a priori indices and colorectal cancer risk by sex and race/ethnicity: the Multiethnic Cohort Study

**DOI:** 10.1186/s12916-022-02623-7

**Published:** 2022-11-29

**Authors:** Jihye Kim, Carol J. Boushey, Lynne R. Wilkens, Christopher A. Haiman, Loïc Le Marchand, Song-Yi Park

**Affiliations:** 1 Department of Genetics and Biotechnology, College of Life Sciences, Kyung Hee University, 1732 Deogyeong-daero, Giheung-Gu, Yongin, Gyeonggi-Do 17104 South Korea; 2 Cancer Epidemiology Program, University of Hawaii Cancer Center, Honolulu, HI USA; 3 Department of Population and Public Health Sciences, Keck School of Medicine and Norris Comprehensive Cancer Center, University of Southern California, Los Angeles, CA USA

**Keywords:** Plant-based diets, Colorectal cancer, Plant food quality, Multiethnic cohort

## Abstract

**Background:**

Plant-based diets assessed by a priori indices are associated with health outcomes. This study investigated the associations between pre-defined indices of plant-based diets and risk of colorectal cancer (CRC) and evaluated whether the association varies by sex, race and ethnicity, and anatomic subsite of tumors.

**Methods:**

A total of 79,952 men and 93,475 women who participated in the Multiethnic Cohort Study were included. Primary outcome was incidence of invasive CRC. Cox models were used to estimate the risk of CRC across quintiles of three plant-based diet scores: overall plant-based diet index (PDI), healthful plant-based diet index (hPDI), and unhealthful plant-based diet index (uPDI).

**Results:**

During a mean follow-up of 19.2 years, 4976 incident CRC were identified. Among men, multivariable-adjusted HR (95% CI) for the highest vs. lowest quintiles was 0.77 (0.67–0.88) for PDI, and 0.80 (0.70–0.91) for hPDI, while no significant association was found for uPDI among men and for all indices among women. In men, the inverse association for PDI was stronger in Japanese American, Native Hawaiian, and White groups than African American or Latino group (*P* for heterogeneity = 0.01) and for left colon and rectal tumors than right tumors (*P* for heterogeneity = 0.005), whereas the decreased risk with hPDI was found consistently across racial and ethnic groups and subsites.

**Conclusions:**

Greater adherence to plant-based diets rich in healthy plant foods and low in less healthy plant foods is associated with a reduced risk of CRC in men, but not in women. The strength of the association among men may vary by race and ethnicity and anatomic subsite of tumors.

**Supplementary Information:**

The online version contains supplementary material available at 10.1186/s12916-022-02623-7.

## Background

Colorectal cancer is the third most common malignancy and the fourth most common cause of cancer death worldwide [1]. Although screening and treatment for colorectal cancer have improved, new preventive strategies to lower risk remain a priority. Accumulating evidence indicates that diet is an important modifiable risk factor for colorectal cancer. Red and processed meats are associated with an increased risk [2–4], whereas foods rich in dietary fiber are associated with a decreased risk of colorectal cancer [4–6], suggesting that plant-based diets play a role in the prevention of the disease. Plant-based diets include various dietary patterns, defined in terms of low consumption of animal foods [7]. Vegetarian or vegan diet excludes some or all animal foods. Lacto-(ovo)-vegetarians or pesco-vegetarians consume dairy foods/egg or additionally fish. However, the definitions of vegan or vegetarian diets do not consider the nutritional quality of plant foods, despite the fact that not all plant foods are healthy. For instance, some plant foods, such as refined grains, sweets, and sugar-sweetened beverages adversely affect colorectal cancer incidence [8, 9].

Recently, a priori plant-based diet indices have been developed to assess intakes of both plant and animal foods, considering the quality of plant foods: overall plant-based diet index (PDI), healthful plant-based diet index (hPDI), and unhealthful plant-based diet index (uPDI). All indices negatively weigh animal foods, but differently weigh plant foods depending on their nutritional quality. Previous studies have reported PDI and hPDI were associated with lower risk of cardiovascular disease, type 2 diabetes, and its related conditions [10–13], whereas uPDI was associated with higher risk of these outcomes [11–14]. Yet, it remains unknown whether these indices are associated with colorectal cancer risk, especially in racially and ethnically diverse populations.

We evaluated the associations between the three indices of plant-based diets and risk of colorectal cancer in the Multiethnic Cohort (MEC) Study and whether the associations varied by sex, race and ethnicity, and anatomical subsite of tumors.

## Methods

### Study population

The MEC is a population-based, prospective cohort study designed to investigate lifestyle and genetic factors related to cancer and other chronic diseases [15]. Participants were identified from the sources including driver’s license records, voter registration lists, and Health Care Financing Administration data files. They were recruited by mailing an invitation letter and questionnaire [15]. More than 215,000 adults aged 45–75 years living in Hawaii or the Los Angeles area were enrolled between 1993 and 1996. Participants were primarily African American, Japanese American, Native Hawaiian, Latino, or White. The response rates from highest to lowest were Japanese American men 46%, women 51%; White men 39%, women 47%; Native Hawaiian men 36%, women 42%; African American men 20%, women 26%; and Latino men 19%, women 21%. The respondents completed a self-administered, comprehensive questionnaire including a detailed dietary assessment. The Institutional Review Boards of the University of Hawaii and the University of Southern California approved the study.

For the current analysis, we excluded participants who were not of one of the main five racial and ethnic groups (*n* = 13,987), had colorectal cancer prior to baseline based on self-report (*n* = 2251) or tumor registry information (*n* = 300), and reported implausible diets (*n* = 8,137). Specifically, we computed a robust standard deviation (RSD) with an assumption of a truncated normal distribution from the middle 80% of the log energy distribution. Then, we excluded all individuals with energy values out of the ranges of means ± 3RSD. A similar approach was used to exclude individuals with extreme fat, protein, or carbohydrate intakes to identify individuals who skipped important dietary pages [16]. As a result, the range of total energy intake after exclusions was 490 to 8700 kcal/day for men and 425 to 7800 kcal/day for women. We further excluded participants with missing covariates (*n* = 19,234) including body mass index (BMI), smoking, physical activity, multivitamin use, nonsteroidal anti-inflammatory drug (NSAID) use, and menopausal hormone therapy use for women. As a result, a total of 79,952 men and 93,475 women were included in the analysis.

### Dietary assessment and plant-based diet indices

At baseline, participants’ usual intake of foods and beverages was assessed with a quantitative food frequency questionnaire (QFFQ) with > 180 food items [15]. Participants reported the frequency and the usual portion size of food consumption in the previous year. The QFFQ had 8 response categories (“never or hardly ever” to “2 or more times a day”) and, for some beverage items, 9 response categories (“never or hardly ever” to “4 or more times a day”). Participants were asked to choose one of three (in a few instances four) portion size options specific to each food item to assess quantity of food eaten. The QFFQ was validated in all sex-ethnic groups in a calibration study with the use of data from three repeated 24-h dietary recalls [17]. Daily energy and nutrient intakes were calculated using the food composition tables developed by the University of Hawaii Cancer Center for use in the MEC.

We calculated three plant-based diet indices (PDI, hPDI, and uPDI) using data from the QFFQ, based on the food groups defined and the scoring methods developed in previous studies [12, 13, 18]. For the current study, 16 food groups were used for the PDI, hPDI, and uPDI. The food groups were classified as healthy plant foods (whole grains, fruits, vegetables, vegetable oils, nuts, legumes, tea and coffee), less healthy plant foods (refined grains, fruit juices, potatoes, added sugars), and animal foods (animal fat, dairy, eggs, fish or seafood, meat) for PDI, hPDI, and uPDI based on the associations between food items and health outcomes reported in the literature [12, 13]. We modified the original 18 food groups used for PDI, hPDI, and uPDI [12, 13] by combining sugar sweetened beverages and sweets and desserts into added sugars and excluding miscellaneous animal-based foods because we primarily used the MyPyramid Equivalent Database (MPED) values (cup equivalents or ounce equivalents) calculated for the MEC participants [19, 20]. The MPED is a standardized food-grouping system developed by the USDA that disaggregates mixed dishes into their food items and allocates each food item into one of 32 food groups [14]. We used the MPED values for 13 out of 16 component food groups of plant-based diet indices as we did for constructing commonly used diet quality indices from the MEC QFFQ that included many mixed dish items [20]. For vegetable oils, tea and coffee, and animal fat that were not in the MPED groups, gram amounts of individual QFFQ items were used.

For each food group for all indices, daily consumption per 1000 kcal was divided into quintiles based on sex-specific distributions. For the PDI, all plant food groups were positively scored (the lowest quintile receiving 1 point and the highest quintile receiving 5 points). For the hPDI, only healthy plant foods were positively scored, while less healthy plant food groups were reversely scored (the lowest quintile receiving 5 points and the highest quintile receiving 1 point). Conversely, for the uPDI, less healthy plant food groups were positively scored, and healthy plant food groups were reversely scored. In all indices, animal food groups were reversely scored. Higher PDI scores represented greater consumption of all types of plant foods regardless of healthiness. Higher hPDI scores represented greater consumption of healthy plant foods and lower consumption of less healthy plant foods. Higher uPDI score represented lower consumption of healthy plant foods and greater consumption of less healthy plant foods. Total scores for each index were computed as the sum of the scores (1 to 5) across each component food group. Thus, the theoretical range of PDI, hPDI, and uPDI was 16 to 80.

### Cancer ascertainment

Incident colorectal cancer cases were identified by linkage to the statewide Surveillance, Epidemiology, and End Results Program tumor registries in Hawaii and California. Deaths were identified by linkage to death certificate files in both states and the National Death Index. Case and death ascertainment were completed through December 31, 2017. Cases in the current study were limited to invasive adenocarcinoma of the large bowel and were categorized according to anatomic subsites using International Classification of Disease (ICD)-O3 codes: C18.0–C18.5 for right colon, C18.6–C18.7 for left colon, and C19.9 and C20.9 for rectum, excluding multi-site cases. During an average follow-up period of 19.2 years, a total of 4976 incident colorectal cancer cases were identified among the eligible participants.

### Statistical analysis

Cox proportional hazards models of colorectal cancer with age as the time metric were used to calculate hazard ratios (HRs) and 95% confidence intervals (CIs) for men and women separately. Follow-up began at the date of cohort entry and ended at the earliest date of diagnosis, death, or study closure (December 31, 2017). A separate model was fit for each of three diet indices. The total scores for each index were divided into quintiles based on their distributions across the entire cohort. All models were adjusted for race and ethnicity as a strata variable and age at cohort entry (years), family history of colorectal cancer (yes/no), history of colorectal polyp (yes/no), BMI (< 25, 25– < 30, and ≥ 30 kg/m^2^), pack-years of cigarette smoking (continuous), multivitamin use (yes/no), NSAID use (yes/no), physical activity (hours spent in moderate and vigorous work or sports per day), menopausal hormone therapy use (never, past, current) for women only, alcohol consumption (g/day), and total energy intake (log transformed kcal/day) as covariates. We also considered other factors such as height and education levels as covariates but did not include them in the final models because adjustment for these variables did not change the associations between plant-based dietary patterns and colorectal cancer risk. The potential confounders were selected because they were associated with colorectal cancer risk in our cohort or because they were established risk factors for colorectal cancer in the literature. Linear trends were tested by modeling sex- and race- and ethnicity-specific median scores within each quintile as a continuous variable. The proportional hazards assumption was tested by Schoenfeld residual method [21] and found to be met.

Sensitivity analysis was conducted to test the robustness of our findings. We conducted 4-year time-lagged analysis to minimize reverse causation due to existing diseases. To assess the possible impact of residual confounding by the known risk factors of colorectal cancer, we conducted subgroup analyses by BMI (≥ 25 kg/m^2^ vs. < 25 kg/m^2^), smoking status (ever vs. never smokers), and alcohol consumption (≥ 30 g/day vs. < 30 g/day). In addition, we evaluated the associations of the individual plant food groups with colorectal cancer risk and estimated the associations of substituting of whole grains, fruits, vegetables, or legumes for added sugars, which were the major food groups associated with colorectal cancer risk. The substitution analyses were conducted by including both food groups as continuous variables (divided by SD of each variable) in the multivariable model, which also contained total energy intake and other covariates. The difference in their *β* coefficients and their variances and covariance were used to estimate the substitution associations [22]. In supplemental analyses, the plant-based diet indices were updated as time-dependent variables using data from a 10-year follow-up survey (2003–2008) that was available for 79,350 (46%) of the 173,427 participants.

Tests for heterogeneity between subgroups were based on the Wald statistics for cross-product terms of trend variables and subgroup indicator variables (sex or race and ethnicity). Tests for heterogeneity by anatomic subsite were based on the Wald statistics comparing competing risk models using an augmented data approach [23, 24]. Spearman’s correlations were examined between the plant-based diet indices A possible nonlinear relationship between the indices and colorectal cancer risk was examined nonparametrically using restricted cubic splines with 4 knots at 5th, 35th, 65th, and 95th percentiles [25]. All statistical tests were two-sided. All analyses were performed by using SAS statistical software, version 9.4 (SAS Institute, Inc., Cary, NC).

## Results

Baseline characteristics of participants according to the quintiles of plant-based diet indices are shown in Table 1. Men and women in the highest quintiles (Q5) of PDI and hPDI were more likely to be older, to have a history of intestinal polyps, to use multivitamin supplements, to have lower BMI and energy intake, and less likely to be ever smokers, compared with those in the lowest quintile (Q1). Men in Q5 consumed less alcohol than in Q1, with the exception of hPDI. Women in Q5 tended to be more often menopausal hormone therapy users than those in Q1. In contrast, men and women in Q5 of uPDI were more likely to be younger and to have higher energy and alcohol intakes and less likely to have a history of intestinal polyps and to use multivitamin supplements. Women in Q5 of uPDI were less often menopausal hormone therapy users, compared with those in Q1.

**Table 1:** Baseline characteristics of 173,427 participants by lowest (Q1) and highest (Q5) quintiles of 3 plant-based diet indices in the Multiethnic Cohort Study, 1993–1996

	**Total**	**Overall plant-based diet index**		**Healthful plant-based diet index**		**Unhealthful plant-based diet index**	
		Q1	Q5	Q1	Q5	Q1	Q5
**Men**							
No. of participants	79,952	13,902	15,965	16,047	13,573	15,118	15,775
Age at cohort entry, y, mean (SD)	60.0 (8.8)	58.2 (8.7)	61.6 (8.6)	57.3 (8.8)	62.3 (8.4)	61.3 (8.5)	58.4 (8.9)
Race/ethnicity, *n* (%)							
African American	10,381 (13.0)	2345 (16.9)	1679 (10.5)	2425 (15.1)	1433 (10.6)	2256 (14.9)	1668 (10.6)
Japanese American	24,138 (30.2)	3631 (26.1)	5172 (32.4)	4735 (29.5)	4685 (34.5)	3976 (26.3)	5650 (35.8)
Native Hawaiian	5572 (7.0)	1621 (11.7)	625 (3.9)	1758 (11.0)	647 (4.8)	934 (6.2)	1344 (8.5)
Latino	19,198 (24.0)	2973 (21.4)	3930 (24.6)	3092 (19.3)	3158 (23.3)	3382 (22.4)	3562 (22.6)
White	20,663 (25.8)	3332 (24.0)	4559 (28.6)	4037 (25.2)	3650 (26.9)	4570 (30.2)	3551 (22.5)
Family history of colorectal cancer, *n* (%)	5864 (7.3)	977 (7.0)	1238 (7.8)	1152 (7.2)	1035 (7.6)	1164 (7.7)	1091 (6.9)
History of intestinal polyps, *n* (%)	5602 (7.0)	814 (5.9)	1246 (7.8)	926 (5.8)	1118 (8.2)	1150 (7.6)	972 (6.2)
Body mass index, mean (SD), kg/m^2^	26.6 (4.1)	27.2 (4.5)	26.0 (3.6)	27.2 (4.4)	25.9 (3.7)	26.7 (4.1)	26.5 (4.1)
Ever smokers, *n* (%)	55,257 (69.1)	10,390 (74.7)	10,399 (65.1)	11,104 (69.2)	9263 (68.2)	10,604 (70.1)	10,799 (68.5)
Pack-years among ever smokers, mean (SD), year	14.3 (16.8)	23.4 (17.5)	18.4 (15.7)	22.0 (16.9)	19.5 (16.2)	20.2 (16.5)	21.5 (16.8)
Multivitamin use, *n* (%)	37,944 (47.5)	5677 (40.8)	8442 (52.9)	6718 (41.9)	7240 (53.3)	7905 (52.3)	6671 (42.3)
NSAID use, *n* (%)	40,464 (50.6)	6867 (49.4)	8250 (51.7)	7909 (49.3)	6803 (50.1)	8039 (53.2)	7381 (46.8)
Physical activity, mean (SD), hours/day	1.3 (1.5)	1.27 (1.56)	1.41 (1.50)	1.32 (1.55)	1.42 (1.51)	1.38 (1.48)	1.31 (1.53)
Alcohol intake, mean (SD), g/day,	14.8 (32.6)	29.8 (55.2)	7.1 (14.2)	12.0 (23.7)	15.0 (34.5)	9.8 (17.2)	23.2 (52.7)
Energy intake, mean (SD), kcal/day,	2426 (1116)	2584 (1249)	2286 (992)	2599 (1196)	2207 (964)	2300 (1031)	2489 (1143)
**Women**							
No. of participants	93,475	15,371	17,912	19,445	16,773	17,128	19,134
Age at cohort entry, y, mean (SD)	59.3 (8.8)	57.4 (8.7)	61.0 (8.5)	56.6 (8.7)	61.6 (8.3)	60.6 (8.5)	57.9 (8.8)
Race/ethnicity, *n* (%)							
African American	17,248 (18.5)	4019 (26.1)	2434 (13.6)	4195 (21.6)	2573 (15.3)	3488 (20.4)	3230 (16.9)
Japanese American	26,284 (28.1)	2968 (19.3)	6098 (34.0)	4534 (23.3)	5857 (34.9)	4698 (27.4)	5568 (29.1)
Native Hawaiian	6955 (7.4)	1796 (11.7)	768 (4.3)	2213 (11.4)	837 (5.0)	1138 (6.6)	1632 (8.5)
Latino	19,324 (20.7)	2782 (18.1)	4017 (22.4)	3577 (18.4)	3419 (20.4)	2949 (17.2)	4340 (22.7)
White	23,664 (25.3)	3806 (24.8)	4595 (25.7)	4926 (25.3)	4087 (24.4)	4855 (28.3)	4364 (22.8)
Family history of colorectal cancer, *n* (%)	8165 (8.7)	1256 (8.2)	1639 (9.2)	1589 (8.2)	1582 (9.4)	1598 (9.3)	1586 (8.3)
History of intestinal polyps, *n* (%)	4170 (4.5)	602 (3.9)	890 (5.0)	732 (3.8)	796 (4.7)	852 (5.0)	746 (3.9)
Body mass index, mean (SD), kg/m^2^	26.4 (5.5)	27.7 (6.1)	25.2 (4.8)	27.4 (5.9)	25.1 (4.9)	26.3 (5.4)	26.6 (5.7)
Ever smokers, *n* (%)	40,629 (43.5)	7851 (51.1)	6678 (37.3)	8961 (46.1)	6775 (40.4)	7790 (45.5)	8101 (42.3)
Pack-years among ever smokers, mean (SD), year	6.8 (12.3)	17.5 (15.2)	13.6 (13.6)	16.8 (15.0)	14.2 (13.8)	15.1 (14.2)	16.6 (15.1)
Multivitamin use, *n* (%)	50,336 (53.9)	7516 (48.9)	10,431 (58.2)	9404 (48.4)	9920 (59.1)	10,016 (58.5)	9293 (48.6)
NSAID use, *n* (%)	49,543 (53.0)	8784 (57.1)	8927 (49.8)	10,903 (56.1)	8066 (48.1)	9138 (53.4)	9921 (51.9)
MHT ever use, *n* (%)	44,051 (47.1)	6471 (42.1)	9098 (50.8)	8073 (41.5)	8745 (52.1)	8668 (50.6)	8276 (43.3)
Physical activity, mean (SD), hours/day	1.1 (1.3)	0.99 (1.21)	1.21 (1.29)	1.02 (1.21)	1.23 (1.30)	1.21 (1.28)	1.02 (1.22)
Alcohol intake, mean (SD), g/day	4.4 (14.9)	8.3 (25.1)	2.4 (7.3)	4.3 (13.4)	3.8 (13.7)	3.6 (8.8)	5.9 (23.6)
Energy intake, mean (SD), kcal/day,	1973 (945)	2032 (1049)	1906 (839)	2124 (1050)	1825 (809)	1876 (849)	2024 (1004)

*SD* Standard deviation, *NSAID* Nonsteroidal anti-inflammatory drug, *MHT* Menopausal hormone therapy

Since all indices were based on sex-specific quintiles of food group intakes, the mean scores were very similar between men and women. In both men and women, the mean scores of PDI and hPDI were highest in Japanese American group (49.2 and 49.0 in men, 49.7 and 49.6 in women) and lowest in Native Hawaiian (46.7 and 46.8 in men, 47.0 and 46.8 in women) group. The mean uPDI was highest in Native Hawaiian men (49.3) and women (49.4) and lowest in African American men (47.9) and White women (48.3) (Additional file 1: Table S1). The three plant-based diet indices were moderately to strongly correlated with each other except for uPDI with PDI (Additional file 1: Table S2). PDI showed the highest correlation with hPDI ($\rho$ = 0.60 and 0.61), while the correlations ranged from -0.36 (men) to -0.39 (women) between hPDI and uPDI in both sexes.

**Fig. 1:**
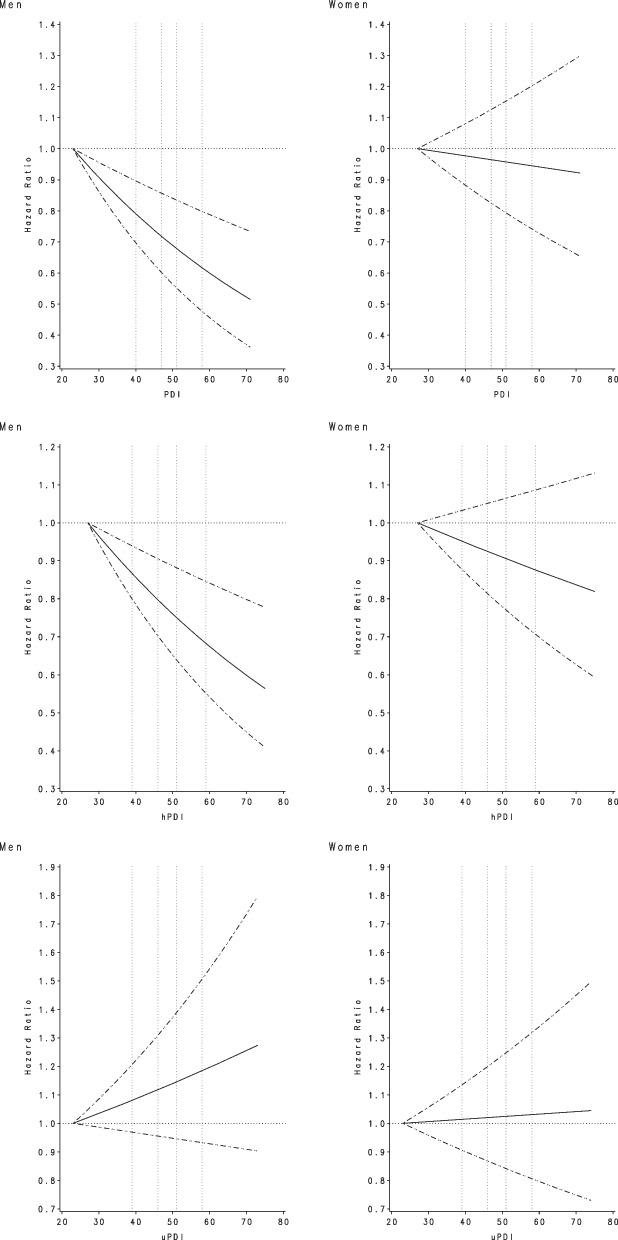
Association between plant-based diet indices and colorectal cancer risk, based on restricted cubic splines, among men and women in the Multiethnic Cohort Study, 1993–2017. The solid line indicates the hazard ratio, and the dashed lines indicate the 95% confidence intervals for adjusted estimates. The 4 knots are shown as the vertical dashed lines at 5th, 35th, 65th, and 95th percentiles. Models were adjusted for age at cohort entry, race and ethnicity, family history of colorectal cancer, history of colorectal polyp, body mass index, pack-years of cigarette smoking, multivitamin use, nonsteroidal anti-inflammatory drug use, physical activity, menopausal hormone therapy use for women only, alcohol consumption, and total energy intake. PDI, overall plant-based diet index; hPDI, healthful plant-based diet index; uPDI, unhealthful plant-based diet index

Plant-based diet indices were significantly inversely associated with risk of colorectal cancer in men, but not in women (Table 2). Men in Q5 of PDI and hPDI had a 24% (HR = 0.76, 95% CI: 0.67–0.87) and 21% (HR = 0.79, 95% CI: 0.69–0.91) lower risk of colorectal cancer, compared to those in Q1 of each index, respectively (all *P* for trend < 0.001), while no significant association was found for uPDI. For women, none of the plant-based diet indices was significantly associated with colorectal cancer risk (*P* for heterogeneity by sex ≤ 0.05 for PDI and hPDI). In the sensitivity analysis excluding the cases diagnosed within the first 4 years of follow-up, the findings remained similar in men (Q5 vs Q1: HR = 0.74, 95% CI: 0.64–0.86 for PDI, HR = 0.78, 95% CI: 0.67–0.91 for hPDI, and HR = 1.11, 95% CI: 0.97–1.27 for uPDI). Restricted cubic splines showed no evidence of nonlinearity for the significant associations of PDI and hPDI with colorectal cancer risk in men (all *P* for linearity < 0.001) (Fig. 1).

**Table 2:** Hazard ratios (95% confidence intervals) for colorectal cancer risk according to plant-based diet indices in the Multiethnic Cohort Study, 1993–2017

	**Men (*n* = 79,952)**		**Women (*n* = 93,475)**		***P* for heterogeneity by sex**
	**No. of cases**	**HR (95% CI)**^**a**^	**No. of cases**	**HR (95% CI)**^**a**^	
**Overall plant-based diet index**					
Q1 (23–43)	487	1.00 (ref)	385	1.00 (ref)	
Q2 (44–47)	583	0.89 (0.79–1.01)	556	0.98 (0.86–1.12)	
Q3 (48–50)	579	0.95 (0.84–1.07)	535	0.99 (0.86–1.13)	
Q4 (51–53)	475	0.89 (0.78–1.01)	424	0.88 (0.77–1.02)	
Q5 (54–71)	458	0.76 (0.67–0.87)	494	0.99 (0.86–1.14)	
P for trend		< 0.001		0.53	0.05
**Healthful plant-based diet index**					
Q1 (27–43)	518	1.00 (ref)	455	1.00 (ref)	
Q2 (44–47)	614	0.95 (0.84–1.06)	533	0.97 (0.85–1.10)	
Q3 (48–50)	532	0.96 (0.85–1.08)	486	1.06 (0.93–1.20)	
Q4 (51–54)	500	0.85 (0.75–0.96)	475	0.93 (0.81–1.06)	
Q5 (55–75)	418	0.79 (0.69–0.91)	445	0.91 (0.80–1.04)	
P for trend		0.0001		0.14	0.047
**Unhealthful plant-based diet index**					
Q1 (23–43)	497	1.00 (ref)	472	1.00 (ref)	
Q2 (44–47)	594	0.93 (0.83–1.05)	568	0.96 (0.85–1.09)	
Q3 (48–50)	512	0.97 (0.86–1.10)	477	0.96 (0.85–1.09)	
Q4 (51–53)	424	0.96 (0.84–1.09)	398	0.97 (0.85–1.11)	
Q5 (54–74)	555	1.08 (0.95–1.22)	479	1.01 (0.89–1.15)	
P for trend		0.19		0.85	0.24

^a^Models were adjusted for age at cohort entry, race and ethnicity, family history of colorectal cancer, history of colorectal polyp, body mass index, pack-years of cigarette smoking, multivitamin use, nonsteroidal anti-inflammatory drug use, physical activity, menopausal hormone therapy use for women only, alcohol consumption, and total energy intake

In the subgroup analyses by BMI, smoking status, and alcohol consumption, the results remained similar across the subgroups for men. However, in women, we found a significant inverse association for hPDI in ever smokers (Q5 vs Q1: HR = 0.80, 95% CI: 0.65–0.98), but not in never smokers (Additional file 1: Table S3). We compared daily consumption of component food groups in the lowest, middle, and highest quintiles of each plant-based diet index by sex (Additional file 1: Table S4). Women consumed higher amounts of healthy plant foods and lower amounts of less healthy plant foods than did men.

The analysis of individual food components showed that higher intake of whole grains (HR per SD = 0.95, 95% CI: 0.91–0.995) and legumes (HR per SD = 0.91, 95% CI: 0.87–0.96) were associated with lower risk of colorectal cancer (Additional file 1: Table S5). In contrast, higher intake of added sugar was associated with higher risk of colorectal cancer although it did not reach significance (HR per SD = 1.03, 95% CI: 0.99–1.08). In substitution analyses in which two SD of added sugars were replaced by whole grains, fruits, vegetables, or legumes, we observed a 9% (HR = 0.91, 95% CI: 0.84–0.995), 8% (HR = 0.92, 95% CI: 0.84–1.00), 8% (HR = 0.93, 95% CI: 0.85–1.01), or 12% (HR = 0.88, 95% CI: 0.80–0.96) reduction in risk of colorectal cancer, respectively.

In race- and ethnicity-specific analysis (Table 3), an inverse association for PDI was significant for Japanese American and White men and suggested for Native Hawaiian men (*P* for heterogeneity = 0.01 across 5 groups; < 0.001 between Japanese American, Native Hawaiian, and White groups combined vs. African American and Latino groups combined). For hPDI, all HRs for Q5 were below 1 (*P* for heterogeneity = 0.91), although only in Japanese American and White groups did the decreasing trend reach statistical significance. No significant association was found for uPDI in any racial and ethnic groups (*P* for heterogeneity = 0.91). Among women, none of the racial and ethnic groups showed a significant association for any index.

**Table 3:** Hazard ratios (95% confidence intervals) for colorectal cancer risk with plant-based diet indices by race and ethnicity in the Multiethnic Cohort Study, 1993–2017

	**African American**		**Japanese American**		**Native Hawaiian**		**Latino**		**White**		***P* for heterogeneity**
	**Cases**	**HR (95% CI) **^**a**^	**Cases**	**HR (95% CI) **^**a**^	**Cases**	**HR (95% CI) **^**a**^	**Cases**	**HR (95% CI) **^**a**^	**Cases**	**HR (95% CI) **^**a**^	
**Men**		**(*n* = 10,381)**		**(*n* = 24,138)**		**(*n* = 5572)**		**(*n* = 19,198)**		**(*n* = 20,663)**	
**Overall plant-based diet index**											
Q1	74	1.00 (ref)	173	1.00 (ref)	55	1.00 (ref)	90	1.00 (ref)	95	1.00 (ref)	
Q2	85	0.99 (0.72–1.36)	205	0.84 (0.68–1.03)	48	0.99 (0.67–1.46)	122	0.88 (0.67–1.16)	123	0.95 (0.72–1.24)	
Q3	66	0.93 (0.66–1.30)	220	0.90 (0.74–1.11)	35	0.92 (0.60–1.42)	135	1.03 (0.78–1.35)	123	0.99 (0.75–1.31)	
Q4	61	1.04 (0.73–1.47)	188	0.87 (0.70–1.08)	20	0.76 (0.45–1.28)	109	0.95 (0.71–1.26)	97	0.84 (0.62–1.12)	
Q5	70	1.16 (0.83–1.63)	173	0.70 (0.56–0.87)	14	0.59 (0.32–1.08)	114	0.88 (0.66–1.16)	87	0.65 (0.48–0.88)	
P for trend		0.39		0.005		0.09		0.54		0.004	0.01
**Healthful plant-based diet index**											
Q1	95	1.00 (ref)	177	1.00 (ref)	55	1.00 (ref)	86	1.00 (ref)	105	1.00 (ref)	
Q2	84	0.77 (0.57–1.03)	215	0.99 (0.81–1.20)	47	1.00 (0.68–1.48)	141	1.03 (0.79–1.35)	127	0.93 (0.72–1.21)	
Q3	59	0.70 (0.51–0.97)	206	1.09 (0.89–1.34)	31	0.93 (0.59–1.46)	135	1.07 (0.82–1.41)	101	0.85 (0.64–1.12)	
Q4	65	0.77 (0.56–1.06)	188	0.89 (0.72–1.10)	21	0.73 (0.43–1.22)	120	0.92 (0.70–1.22)	106	0.81 (0.61–1.06)	
Q5	53	0.81 (0.58–1.14)	173	0.80 (0.65–1.00)	18	0.76 (0.43–1.32)	88	0.86 (0.64–1.16)	86	0.76 (0.57–1.01)	
P for trend		0.16		0.03		0.19		0.19		0.03	0.91
**Unhealthful plant-based diet index**											
Q1	74	1.00 (ref)	172	1.00 (ref)	35	1.00 (ref)	107	1.00 (ref)	109	1.00 (ref)	
Q2	91	1.01 (0.74–1.37)	212	0.91 (0.75–1.12)	37	0.80 (0.50–1.27)	129	0.85 (0.66–1.10)	125	1.01 (0.78–1.31)	
Q3	70	1.02 (0.74–1.42)	175	0.87 (0.70–1.07)	27	0.65 (0.40–1.08)	130	1.01 (0.78–1.30)	110	1.19 (0.91–1.55)	
Q4	54	1.01 (0.71–1.44)	175	0.96 (0.78–1.19)	28	0.76 (0.46–1.26)	81	0.77 (0.57–1.02)	86	1.17 (0.88–1.55)	
Q5	67	1.23 (0.88–1.74)	225	0.98 (0.80–1.20)	45	0.94 (0.60–1.48)	123	1.11 (0.85–1.45)	95	1.15 (0.87–1.53)	
P for trend		0.30		0.95		0.82		0.58		0.17	0.91
**Women**		**(*n* = 17,248)**		**(*n* = 26,284)**		**(*n* = 6955)**		**(*n* = 19,324)**		**(*n* = 23,664)**	
**Overall plant-based diet index**											
Q1	120	1.00 (ref)	90	1.00 (ref)	35	1.00 (ref)	56	1.00 (ref)	84	1.00 (ref)	
Q2	145	1.02 (0.80–1.31)	167	0.96 (0.74–1.24)	34	0.95 (0.59–1.54)	88	0.95 (0.68–1.33)	122	0.96 (0.73–1.27)	
Q3	108	0.90 (0.69–1.17)	158	0.81 (0.62–1.05)	38	1.45 (0.91–2.32)	104	1.18 (0.85–1.64)	127	1.06 (0.81–1.41)	
Q4	68	0.74 (0.54–1.00)	168	0.90 (0.69–1.17)	23	1.23 (0.72–2.11)	76	0.93 (0.65–1.31)	89	0.83 (0.61–1.13)	
Q5	90	1.03 (0.78–1.37)	201	0.94 (0.73–1.21)	16	1.22 (0.67–2.26)	90	1.03 (0.74–1.45)	97	0.91 (0.67–1.22)	
P for trend		0.45		0.73		0.23		0.89		0.34	0.94
**Healthful plant-based diet index**											
Q1	123	1.00 (ref)	115	1.00 (ref)	39	1.00 (ref)	76	1.00 (ref)	102	1.00 (ref)	
Q2	131	1.00 (0.78–1.28)	153	0.96 (0.75–1.22)	39	1.31 (0.84–2.06)	93	0.88 (0.65–1.20)	117	0.93 (0.72–1.22)	
Q3	113	1.07 (0.82–1.38)	147	1.01 (0.79–1.29)	27	1.33 (0.80–2.20)	89	1.00 (0.74–1.36)	110	1.08 (0.83–1.42)	
Q4	84	0.78 (0.59–1.03)	178	0.99 (0.78–1.26)	24	1.24 (0.74–2.10)	87	0.89 (0.66–1.22)	102	0.92 (0.70–1.21)	
Q5	80	0.86 (0.65–1.15)	191	0.96 (0.76–1.22)	17	1.20 (0.67–2.18)	69	0.80 (0.57–1.11)	88	0.89 (0.67–1.20)	
P for trend		0.13		0.83		0.44		0.23		0.47	0.92
**Unhealthful plant-based diet index**											
Q1	111	1.00 (ref)	156	1.00 (ref)	20	1.00 (ref)	74	1.00 (ref)	111	1.00 (ref)	
Q2	136	1.03 (0.80–1.32)	189	0.95 (0.77–1.17)	32	1.24 (0.71–2.17)	85	0.81 (0.59–1.10)	126	0.98 (0.76–1.26)	
Q3	108	1.05 (0.81–1.37)	154	0.90 (0.72–1.12)	25	1.07 (0.59–1.93)	77	0.81 (0.59–1.11)	113	1.08 (0.83–1.40)	
Q4	73	0.91 (0.67–1.22)	126	0.91 (0.72–1.16)	25	1.16 (0.64–2.10)	92	1.07 (0.78–1.45)	82	0.97 (0.73–1.29)	
Q5	103	1.13 (0.86–1.49)	159	0.98 (0.78–1.22)	44	1.62 (0.94–2.79)	86	0.84 (0.61–1.14)	87	0.95 (0.72–1.26)	
P for trend		0.60		0.76		0.10		0.75		0.79	0.24

^a^Models were adjusted for age at cohort entry, family history of colorectal cancer, history of colorectal polyp, body mass index, pack-years of cigarette smoking, multivitamin use, nonsteroidal anti-inflammatory drug use, physical activity, menopausal hormone therapy use for women only, alcohol consumption, and total energy intake

For anatomic subsite-specific analysis (Table 4) in men, the inverse association of PDI was stronger for left colon and rectum tumors than for right colon tumor (*P* for heterogeneity = 0.005), while the risk reduction with hPDI was significant for all subsites (*P* for heterogeneity = 0.16). uPDI was associated with increased risk of rectal cancer, but not right or left colon cancer in men (*P* for heterogeneity = 0.048). Among women, no significant association was observed across the subsites of tumors.

**Table 4:** Hazard ratios (95% confidence intervals) for colorectal cancer risk with plant-based diet indices by anatomic subsite in the Multiethnic Cohort Study, 1993–2017

	**Right colon**		**Left colon**		**Rectum**		***P* for heterogeneity**
	**Cases**	**HR (95% CI) **^**a**^	**Cases**	**HR (95% CI) **^**a**^	**Cases**	**HR (95% CI) **^**a**^	
**Men**							
**Overall plant-based diet index**							
Q1	185	1.00 (ref)	149	1.00 (ref)	140	1.00 (ref)	
Q2	244	0.96 (0.79–1.16)	164	0.84 (0.67–1.06)	159	0.87 (0.69–1.09)	
Q3	235	0.97 (0.79–1.18)	178	0.99 (0.79–1.24)	146	0.86 (0.68–1.09)	
Q4	198	0.92 (0.75–1.13)	132	0.85 (0.67–1.08)	137	0.94 (0.73–1.20)	
Q5	221	0.90 (0.73–1.10)	107	0.62 (0.48–0.81)	112	0.69 (0.53–0.89)	
P for trend		0.27		0.002		0.02	0.005
**Healthful plant-based diet index**							
Q1	210	1.00 (ref)	152	1.00 (ref)	143	1.00 (ref)	
Q2	253	0.93 (0.77–1.12)	177	0.94 (0.75–1.17)	164	0.94 (0.75–1.18)	
Q3	211	0.90 (0.74–1.09)	163	1.02 (0.81–1.27)	149	1.01 (0.80–1.28)	
Q4	226	0.90 (0.74–1.09)	122	0.72 (0.56–0.91)	137	0.88 (0.70–1.12)	
Q5	183	0.81 (0.66–0.99)	116	0.77 (0.60–0.99)	101	0.74 (0.57–0.96)	
P for trend		0.04		0.007		0.02	0.16
**Unhealthful plant-based diet index**							
Q1	221	1.00 (ref)	147	1.00 (ref)	112	1.00 (ref)	
Q2	267	0.96 (0.80–1.15)	152	0.80 (0.64–1.00)	159	1.07 (0.84–1.37)	
Q3	213	0.94 (0.78–1.14)	151	0.95 (0.76–1.20)	134	1.08 (0.84–1.39)	
Q4	170	0.91 (0.74–1.11)	112	0.83 (0.65–1.07)	131	1.23 (0.95–1.58)	
Q5	212	1.02 (0.84–1.24)	168	1.04 (0.83–1.31)	158	1.24 (0.97–1.59)	
P for trend		0.98		0.60		0.05	0.048
**Women**							
**Overall plant-based diet index**							
Q1	197	1.00 (ref)	99	1.00 (ref)	72	1.00 (ref)	
Q2	294	1.00 (0.84–1.20)	142	0.98 (0.76–1.27)	100	0.96 (0.71–1.31)	
Q3	272	0.96 (0.80–1.16)	126	0.92 (0.71–1.21)	116	1.18 (0.88–1.60)	
Q4	242	0.96 (0.79–1.17)	113	0.94 (0.72–1.24)	59	0.69 (0.48–0.98)	
Q5	265	1.01 (0.83–1.22)	125	1.01 (0.77–1.33)	89	1.00 (0.72–1.38)	
P for trend		0.96		0.97		0.52	0.38
**Healthful plant-based diet index**							
Q1	227	1.00 (ref)	129	1.00 (ref)	82	1.00 (ref)	
Q2	290	1.04 (0.87–1.24)	124	0.82 (0.64–1.05)	101	1.03 (0.77–1.38)	
Q3	251	1.06 (0.89–1.27)	117	0.93 (0.72–1.20)	103	1.28 (0.95–1.72)	
Q4	255	0.96 (0.80–1.16)	124	0.89 (0.69–1.15)	74	0.83 (0.60–1.14)	
Q5	247	0.97 (0.81–1.17)	111	0.84 (0.65–1.10)	76	0.91 (0.66–1.25)	
P for trend		0.59		0.36		0.30	0.27
**Unhealthful plant-based diet index**							
Q1	251	1.00 (ref)	122	1.00 (ref)	80	1.00 (ref)	
Q2	306	0.99 (0.83–1.16)	141	0.91 (0.71–1.16)	96	0.95 (0.70–1.28)	
Q3	264	1.02 (0.86–1.21)	115	0.87 (0.67–1.12)	83	0.96 (0.71–1.31)	
Q4	210	0.99 (0.83–1.19)	96	0.87 (0.66–1.14)	76	1.06 (0.78–1.46)	
Q5	239	0.99 (0.83–1.19)	131	1.00 (0.78–1.28)	101	1.19 (0.89–1.61)	
P for trend		0.98		0.88		0.17	0.22

^a^Models were adjusted for age at cohort entry, race and ethnicity, family history of colorectal cancer, history of colorectal polyp, body mass index, pack-years of cigarette smoking, multivitamin use, nonsteroidal anti-inflammatory drug use, physical activity, menopausal hormone therapy use for women only, alcohol consumption, and total energy intake

## Discussion

In this large multiethnic population, greater adherence to overall or healthful plant-based diet assessed by a priori indices was associated with lower risk of colorectal cancer, but only in men. The inverse association of overall plant-based diet among men was greater in Japanese American, Native Hawaiian, and White than in African American and Latino groups, and for left colon and rectum tumor than for right colon tumor, whereas the decreased risk with healthful plant-based diet was suggested across racial and ethnic groups and observed for all tumor subsites. Unhealthful plant-based diet showed a trend of increased risk for tumors of the rectum, but not the right or left colon, among men. These findings emphasize the potential importance of the quality of plant foods on the prevention of colorectal cancer and suggest that the benefits from plant-based diets may vary by sex, race and ethnicity, and anatomic subsite of tumor.

Previous prospective studies on plant-based diets have shown inconsistent results. In the NIH-AARP Diet and Health Study, a vegetable and fruit pattern defined a posteriori using a cluster analysis was associated with a 15% reduced risk of colorectal cancer only in men [26]. In the UK Biobank study, low meat-eaters had a 9% lower risk of colorectal cancer in comparison to regular meat-eaters only in men [27]. However, in European cohorts, no association with colorectal cancer risk was found for vegetarians compared with meat eaters or nonvegetarians [28]. These studies dichotomously defined vegetarian diets based on meat consumption from food frequency questionnaires without considering the quality of plant foods. To the best of our knowledge, our study is the first attempt to provide data on the association between plant-based dietary patterns reflecting the quality of plant foods and risk of colorectal cancer.

Several mechanisms can be speculated by which healthful plant-based diets lower risk of colorectal cancer. A variety of healthy plant foods are included in the indices such as fruits, vegetables, and whole grains that are rich in dietary fiber [5, 6], polyphenols [29, 30], or carotenoids [31] with antioxidant and anti-inflammatory features. It is also postulated that gut microbiota may play a mediating role for decreasing risk of colorectal cancer by healthy plant foods [32]. For instance, dietary fiber leads to production of short-chain fatty acids through microbial fermentation, which maintain mucosal integrity and suppresses inflammation and carcinogenesis through effects on immunity and gene expression [33, 34]. In addition, lower amounts of unhealthy plant foods and animal foods in healthful plant-based diet are likely attributable to the reduction in risk of colorectal cancer. Refined grains and added sugars [8, 9] may increase hyperinsulinemia [35, 36] and red and processed meats are associated with the production of genotoxic free radicals and lipid peroxidation [2], which have been proposed to increase colorectal cancer risk.

In the current study, the association of plant-based diet indices with a lower risk of colorectal cancer was observed only in men. The sex differences may be attributable to different dietary habits between men and women. Women consume more plant foods and less animal foods compared to men in general. In our study population, women consumed greater amounts of healthy plant foods and less amounts of unhealthy plant foods compared to men, and they might not have further benefits with higher scores of plant-based diet indices. In addition, men are at higher risk for colorectal cancer than women in general [37] and in the MEC [38], and thus plant-based diets may provide more benefits in reducing risk for them than for women. As another potential explanation for the sex differences, we considered menopausal hormone therapy use, which was associated with a lower risk of colorectal cancer among postmenopausal women in the MEC [39]. However, when stratifying the analysis by menopausal hormone therapy use, we found no significant associations between PDI and colorectal cancer in either users or non-users.

Interestingly, the inverse association between overall plant-based diet and colorectal cancer among men was stronger in Japanese American and White groups than in African American group, which was consistent with our findings on diet quality in the MEC [16]. This pattern of association may be attributable to the differences in non-dietary lifestyle risk factors among racial and ethnic groups. In the MEC, African American men had higher rates of obesity and smoking and less physical activity than did Japanese American and White men. Further research on potential interactions between genetic and environmental factors are required to elucidate racial and ethnic differences in the diet-colorectal cancer relationship.

The decrease in risk of colorectal cancer with overall plant-based diet was greater for left colon and rectum tumor than for right tumor among men in this study. Similarly, prior studies showed that the inverse associations of whole grain, vegetables, or cereal fiber with cancer risk increased from the cecum to rectum suggesting the intricate interplay of diet, gut microbiota, and colorectal cancer [40, 41]. Moreover, compared to the right colon, the left colon and rectum are much more exposed to genotoxic and cytotoxic damages due to the longer transit time and to the fecal mass storage before elimination through defecation [41, 42]. Nevertheless, in the present study, the inverse association of the healthful plant-based diet index was suggested in all racial and ethnic groups and observed for all subsites of tumor among men, which emphasizes the role of the quality of plant foods in plant-rich diets for colorectal cancer prevention.

Strengths of the current study include the prospective design with a large sample size, racial and ethnic diversity of the study population, large number of colorectal cancer cases with a long follow-up period, and comprehensive information on potential confounders. In addition, food group consumption was primarily based on the MyPyramid Equivalent Database, which disaggregates mixed dishes into ingredients. Therefore, classification of foods (16 or 11 groups) and food groups (healthy plant, less healthy plant, or animal) should be reasonably complete for the score calculation.

However, several limitations should be noted. Selection bias as a result of varying response rates across racial and ethnic groups may limit external validity of our findings to general populations. All animal foods were scored negatively in the calculation of plant-based diet indices, while certain animal foods such as fish and dairy foods may have either beneficial or null effects to colorectal cancer. Based on the Third Expert Report by the World Cancer Research Fund and the American Institute for Cancer Research, dairy products including milk probably decrease risk of colorectal cancer in a dose–response manner [4]. In the Adventist Health Study-2, pesco-vegetarians had the lowest risk of colorectal cancer compared with vegan, lacto-ovovegetarian, and semi-vegetarian group [43] supporting protective effects of fish and dairy foods. It might be helpful to consider the healthiness and quality of animal foods for the future research on the association of plant-based diets with colorectal cancer risk. Residual or unmeasured confounding might still exist despite the adjustment for most important risk factors for colorectal cancer. However, the subgroup analyses suggest that the impact of residual confounding due to BMI, smoking status, and alcohol consumption was minimal. The current analysis was based on diet measured at baseline only, although dietary habits might change over time. When updating dietary information using data from the 10-year follow-up QFFQ, the main findings remained similar. However, the data was only available for 46% of the participants. Lastly, some risk estimates for subgroups especially Native Hawaiians should be interpreted with caution due to the smaller sample size.

## Conclusions

In a large multiethnic population, plant-based diets especially rich in healthy plant foods and low in less healthy plant foods were associated with a lower risk of colorectal cancer in men but not in women and the strength of the associations among men varied by race and ethnicity and anatomic subsite of tumors. Our findings support that improving the quality of plant foods and reducing animal food consumption can help prevent colorectal cancer.

## Supplementary Information

**Additional file 1: Table S1.** Mean scores of plant-based diet indices by sex and race and ethnicity. **Table S2.** Correlation coefficients between plant-based diet indices. **Table S3.** Hazard ratios (95% confidence intervals) for colorectal cancer in the highest vs. lowest quintiles of plant-based diet indices in subgroups. **Table S4.** Consumption of component food groups by quintiles of plant-based diet indices by sex. **Table S5.** Hazard ratios (95% confidence intervals) for colorectal cancer according to individual plant food consumption and substitution analysis in men.

## Data Availability

No additional data available. The lead author (JK) affirms that the manuscript is an honest, accurate, and transparent account of the study being reported; that no important aspects of the study have been omitted; and that any discrepancies from the study as planned (and, if relevant, registerd) have been explained.

## Abbreviations

BMI: Body mass index
CRC: Colorectal cancer
CI: Confidence interval
HR: Hazard ratio
hPDI: Healthful plant-based diet index
ICD: International Classification of Disease
MEC: Multiethnic cohort
MPED: MyPyramid equivalent database
NSAID: Nonsteroidal anti-inflammatory drug
PDI: Overall plant-based diet index
QFFQ: Quantitative food frequency questionnaire
uPDI: Unhealthful plant-based diet index
