# Supplementary material for: Plant-based dietary patterns defined by a priori indices and colorectal cancer risk by sex and race/ethnicity: the Multiethnic Cohort Study
Source: BMC Med. 2022 Nov 29;20:430. doi: 10.1186/s12916-022-02623-7 (PMC9706862; doi:10.1186/s12916-022-02623-7)
Supplement: Supplementary file 1 — Additional file 1: Table S1. Mean scores of plant-based diet indices by sex and race and ethnicity. Table S2. Correlation coefficients between plant-based diet indices. Table S3. Hazard ratios (95% confidence intervals) for colorectal cancer in the highest vs. lowest quintiles of plant-based diet indices in subgroups. Table S4. Consumption of component food groups by quintiles of plant-based diet indices by sex. Table S5. Hazard ratios (95% confidence intervals) for colorectal cancer according to individual plant food consumption and substitution analysis in men. [file 12916_2022_2623_MOESM1_ESM.docx]

**Table S1. Mean scores of plant-based diet indices by sex and race and ethnicity**

|  | **All** | **African American** | **Japanese American** | **Native Hawaiian** | **Latino** | **White** |
| --- | --- | --- | --- | --- | --- | --- |
| **Men** |  |  |  |  |  |  |
| Overall plant-based diet index | 48.8 (5.5) | 47.8 (5.7) | 49.2 (5.4) | 46.7 (5.5) | 49.0 (5.4) | 49.1 (5.6) |
| Healthful plant-based diet index | 48.7 (6.0) | 47.9 (5.9) | 49.0 (6.3) | 46.8 (6.1) | 49.0 (5.6) | 48.8 (6.1) |
| Unhealthful plant-based diet index | 48.6 (5.8) | 47.9 (5.7) | 49.3 (5.9) | 49.3 (6.0) | 48.6 (5.5) | 48.0 (5.8) |
| **Women** |  |  |  |  |  |  |
| Overall plant-based diet index | 48.8 (5.4) | 47.6 (5.5) | 49.7 (5.1) | 47.0 (5.4) | 49.2 (5.3) | 48.9 (5.4) |
| Healthful plant-based diet index | 48.7 (6.3) | 47.9 (6.1) | 49.6 (6.4) | 46.8 (6.3) | 48.9 (5.9) | 48.6 (6.2) |
| Unhealthful plant-based diet index | 48.8 (5.8) | 48.4 (5.8) | 48.9 (5.9) | 49.4 (5.8) | 49.3 (5.6) | 48.3 (5.8) |

Values are mean (SD).

Plant-based diet index scores are based on sex-specific distributions.

**Table S2. Correlation coefficients between plant-based diet indices***

|  | **Healthful plant-**  **based diet index** | **Unhealthful plant-**  **based diet index** |
| --- | --- | --- |
| **Men** |  |  |
| Overall plant-based diet index | 0.60 | -0.005 |
| Healthful plant-based diet index | - | -0.36 |
| **Women** |  |  |
| Overall plant-based diet index | 0.61 | 0.07 |
| Healthful plant-based diet index | - | -0.39 |

*All values are significant (P<0.001) except for the correlation of PDI and uPDI in men (P=0.20).

**Table S3.** **Hazard ratios (95% confidence intervals) for colorectal cancer in the highest vs. lowest quintiles of plant-based diet indices in subgroups**

|  | **Overall plant-based diet index** | **Healthful plant-based diet index** | **Unhealthful plant-based diet index** |
| --- | --- | --- | --- |
| **Men** |  |  |  |
| **BMI** |  |  |  |
| ≥25 kg/m^2^ | 0.83 (0.70-0.98) | 0.85 (0.72-1.00) | 1.03 (0.88-1.20) |
| <25 kg/m^2^ | 0.66 (0.53-0.83) | 0.71 (0.57-0.89) | 1.17 (0.95-1.44) |
| **Smoking status** |  |  |  |
| Ever smokers | 0.79 (0.67-0.92) | 0.83 (0.71-0.97) | 1.09 (0.94-1.26) |
| Never smokers | 0.70 (0.54-0.90) | 0.70 (0.55-0.90) | 1.06 (0.83-1.34) |
| **Alcohol consumption** |  |  |  |
| ≥30 g/day | 0.55 (0.36-0.85) | 0.64 (0.47-0.88) | 1.15 (0.84-1.57) |
| <30 g/day | 0.83 (0.71-0.96) | 0.82 (0.71-0.95) | 1.08 (0.94-1.24) |
|  |  |  |  |
| **Women** |  |  |  |
| **BMI** |  |  |  |
| ≥25 kg/m^2^ | 1.03 (0.86-1.23) | 0.89 (0.74-1.07) | 1.07 (0.91-1.27) |
| <25 kg/m^2^ | 0.91 (0.73-1.13) | 0.91 (0.74-1.11) | 0.93 (0.76-1.13) |
| **Smoking status** |  |  |  |
| Ever smokers | 1.01 (0.83-1.24) | 0.80 (0.65-0.98) | 1.10 (0.91-1.33) |
| Never smokers | 0.98 (0.81-1.18) | 1.01 (0.84-1.21) | 0.95 (0.80-1.13) |
| **Alcohol consumption** |  |  |  |
| ≥30 g/day | 0.97 (0.48-1.98) | 0.74 (0.38-1.46) | 1.49 (0.74-3.00) |
| <30 g/day | 1.00 (0.87-1.15) | 0.92 (0.80-1.06) | 0.99 (0.87-1.13) |

Models were adjusted for age at cohort entry, race and ethnicity, family history of colorectal cancer, history of colorectal polyp, body mass index, pack-years of cigarette smoking, multivitamin use, nonsteroidal anti-inflammatory drug use, physical activity, menopausal hormone therapy use for women only, alcohol consumption, and total energy intake.

**Table S4. Consumption of component food groups by quintiles of plant-based diet indices by sex**

| **Components** | **Overall plant-based diet index** | | | **Healthful plant-based diet index** | | | **Unhealthful plant-based diet index** | | |
| --- | --- | --- | --- | --- | --- | --- | --- | --- | --- |
|  | Q1 | Q3 | Q5 | Q1 | Q3 | Q5 | Q1 | Q3 | Q5 |
| **Men** |  |  |  |  |  |  |  |  |  |
| **Healthy plant foods** | |  |  |  |  |  |  |  |  |
| Whole grain | 0.5 | 0.7 | 1.0 | 0.4 | 0.7 | 1.2 | 1.0 | 0.7 | 0.5 |
| Fruits | 0.4 | 0.7 | 1.0 | 0.4 | 0.7 | 1.2 | 0.9 | 0.7 | 0.5 |
| Vegetables | 0.7 | 0.9 | 1.1 | 0.7 | 0.9 | 1.2 | 1.2 | 0.9 | 0.7 |
| Vegetable oil | 0.8 | 1.0 | 1.1 | 0.7 | 1.0 | 1.2 | 1.3 | 0.9 | 0.7 |
| Nuts | 0.2 | 0.3 | 0.4 | 0.2 | 0.3 | 0.4 | 0.4 | 0.3 | 0.2 |
| Legumes | 0.1 | 0.1 | 0.1 | 0.1 | 0.1 | 0.1 | 0.1 | 0.1 | 0.1 |
| Coffee/Tea | 148 | 200 | 246 | 150 | 206 | 248 | 245 | 198 | 157 |
| **Less healthy plant foods** | |  |  |  |  |  |  |  |  |
| Refined grains | 2.8 | 2.9 | 2.8 | 3.1 | 2.8 | 2.5 | 2.3 | 2.9 | 3.3 |
| Fruit juices | 0.1 | 0.1 | 0.2 | 0.1 | 0.1 | 0.1 | 0.1 | 0.1 | 0.2 |
| Potatoes | 0.1 | 0.1 | 0.1 | 0.1 | 0.1 | 0.1 | 0.1 | 0.1 | 0.1 |
| Added sugar | 4.7 | 5.1 | 5.2 | 6.1 | 5.0 | 3.9 | 3.7 | 5.0 | 6.9 |
| **Animal foods** |  |  |  |  |  |  |  |  |  |
| Animal fat | 2.9 | 1.6 | 0.6 | 2.9 | 1.5 | 0.6 | 2.5 | 1.6 | 0.9 |
| Dairy | 0.5 | 0.5 | 0.5 | 0.5 | 0.5 | 0.5 | 0.6 | 0.5 | 0.4 |
| Egg | 0.2 | 0.2 | 0.1 | 0.2 | 0.2 | 0.1 | 0.2 | 0.2 | 0.1 |
| Fish/seafood | 0.4 | 0.4 | 0.3 | 0.4 | 0.3 | 0.3 | 0.5 | 0.3 | 0.2 |
| Meat | 2.0 | 1.6 | 1.2 | 1.9 | 1.6 | 1.2 | 1.8 | 1.6 | 1.3 |
| **Women** |  |  |  |  |  |  |  |  |  |
| **Healthy plant foods** | |  |  |  |  |  |  |  |  |
| Whole grain | 0.6 | 0.9 | 1.1 | 0.5 | 0.9 | 1.3 | 1.1 | 0.9 | 0.7 |
| Fruits | 0.7 | 1.0 | 1.4 | 0.6 | 1.0 | 1.6 | 1.2 | 1.0 | 0.8 |
| Vegetables | 0.9 | 1.1 | 1.4 | 0.9 | 1.1 | 1.5 | 1.5 | 1.1 | 0.8 |
| Vegetable oil | 0.8 | 0.9 | 1.0 | 0.7 | 0.9 | 1.0 | 1.2 | 0.9 | 0.7 |
| Nuts | 0.2 | 0.3 | 0.4 | 0.2 | 0.3 | 0.4 | 0.3 | 0.3 | 0.2 |
| Legumes | 0.1 | 0.1 | 0.1 | 0.1 | 0.1 | 0.1 | 0.1 | 0.1 | 0.1 |
| Coffee/Tea | 186 | 250 | 298 | 184 | 257 | 298 | 301 | 246 | 190 |
| **Less healthy plant foods** | |  |  |  |  |  |  |  |  |
| Refined grains | 2.6 | 2.7 | 2.6 | 2.9 | 2.7 | 2.2 | 2.1 | 2.7 | 3.1 |
| Fruit juices | 0.1 | 0.2 | 0.2 | 0.2 | 0.2 | 0.1 | 0.1 | 0.2 | 0.2 |
| Potatoes | 0.1 | 0.1 | 0.1 | 0.1 | 0.1 | 0.1 | 0.1 | 0.1 | 0.1 |
| Added sugar | 4.8 | 5.1 | 5.1 | 6.1 | 5.0 | 3.9 | 3.7 | 4.8 | 6.9 |
| **Animal foods** |  |  |  |  |  |  |  |  |  |
| Animal fat | 3.2 | 1.7 | 0.6 | 2.9 | 1.6 | 0.7 | 2.5 | 1.7 | 1.0 |
| Dairy | 0.7 | 0.6 | 0.5 | 0.6 | 0.6 | 0.5 | 0.7 | 0.6 | 0.5 |
| Egg | 0.2 | 0.2 | 0.1 | 0.2 | 0.2 | 0.1 | 0.2 | 0.2 | 0.1 |
| Fish/seafood | 0.4 | 0.3 | 0.3 | 0.4 | 0.3 | 0.3 | 0.5 | 0.3 | 0.2 |
| Meat | 2.1 | 1.5 | 1.1 | 1.8 | 1.6 | 1.2 | 1.7 | 1.6 | 1.3 |

Values are mean.

Daily intakes are expressed as consumption per 1000 kcal (ounce equivalents for whole grain, refined grain, nuts, egg, fish/seafood, and meat; cup equivalents for fruit, vegetables, legumes, fruit juice, potatoes, and dairy; teaspoons for added sugar; grams for vegetable oil, coffee/tea, and animal fat).

**Table S5**. **Hazard ratios (95% confidence intervals) for colorectal cancer according to individual plant food consumption and substitution analysis in men**

|  | **HR (95% CI)*** | | **SD** |
| --- | --- | --- | --- |
| **Healthy plant foods** |  |  |  |
| Whole grains | 0.95 | (0.91- 0.995) | 0.64 |
| Fruits | 0.96 | (0.92 - 1.00) | 0.61 |
| Vegetables | 0.97 | (0.93 - 1.01) | 0.45 |
| Vegetable oils | 1.02 | (0.98 - 1.06) | 1.29 |
| Nuts | 0.99 | (0.96 - 1.03) | 0.34 |
| Legumes | 0.91 | (0.87 - 0.96) | 0.10 |
| Coffee/Tea | 1.01 | (0.97 - 1.05) | 183 |
| **Less healthy plant foods** |  |  |  |
| Refined grains | 1.00 | (0.96 - 1.05) | 1.04 |
| Fruit juice | 0.96 | (0.92 - 1.00) | 0.18 |
| Potatoes | 1.03 | (0.99 - 1.07) | 0.09 |
| Added sugars | 1.03 | (0.99 - 1.08) | 3.11 |
| **Substitution†** |  |  |  |
| Whole grains for added sugars | 0.91 | (0.84 - 0.995) |  |
| Fruits for added sugars | 0.92 | (0.84 - 1.00) |  |
| Vegetables for added sugars | 0.93 | (0.85 - 1.01) |  |
| Legumes for added sugars | 0.88 | (0.80 - 0.96) |  |

*****HR per SD

**†**Substitute whole grains, fruits, vegetables, or legumes for two SD of added sugars.

Models were adjusted for age at cohort entry, race and ethnicity, family history of colorectal cancer, history of colorectal polyp, body mass index, pack-years of cigarette smoking, multivitamin use, nonsteroidal anti-inflammatory drug use, physical activity, alcohol consumption, total energy intake, and animal food intakes (5 component groups).
